# Multidisciplinary patient care in X‐linked hypophosphatemic rickets: one challenge, many perspectives

**DOI:** 10.1007/s10354-019-00732-2

**Published:** 2020-01-28

**Authors:** Adalbert Raimann, Gabriel T. Mindler, Roland Kocijan, Katrin Bekes, Jochen Zwerina, Gabriele Haeusler, Rudolf Ganger

**Affiliations:** 1 Comprehensive Center for Pediatrics, Department of Pediatrics and Adolescent Medicine, Division of Pediatric Pulmonology, Allergology and Endocrinology, Medical University of Vienna, Vienna, Austria; 2 Department of Pediatric Orthopaedics, Orthopaedic Hospital Speising, Vienna, Austria; 3 Hanusch Hospital of the WGKK and AUVA Trauma Center, 1st Medical Department at Hanusch Hospital, Ludwig Boltzmann Institute of Osteology, Vienna, Austria; 4 Department of Pediatric Dentistry, School of Dentistry, Medical University of Vienna, Vienna, Austria; 5 Vienna Bone and Growth Centre, Vienna, Austria

**Keywords:** XLH, Burosumab, Rare disease, Phosphate, FGF23, Phosphatdiabetes, Burosumab, Seltene Erkrankung, Phosphat, FGF23

## Abstract

X‑linked hypophosphatemic rickets (XLH, OMIM #307800) is a rare genetic metabolic disorder caused by dysregulation of fibroblast-like growth factor 23 (FGF23) leading to profound reduction in renal phosphate reabsorption. Impaired growth, severe rickets and complex skeletal deformities are direct consequences of hypophosphatemia representing major symptoms of XLH during childhood. In adults, secondary complications including early development of osteoarthritis substantially impair quality of life and cause significant clinical burden. With the global approval of the monoclonal FGF23 antibody burosumab, a targeted treatment with promising results in phase III studies is available for children with XLH. Nevertheless, complete phenotypic rescue is rarely achieved and remaining multisystemic symptoms demand multidisciplinary specialist care. Coordination of patient management within the major medical disciplines is a mainstay to optimize treatment and reduce disease burden. This review aims to depict different perspectives in XLH patient care in the setting of a multidisciplinary centre of expertise for rare bone diseases.

## Zusammenfassung

Die X‑chromosomale hypophosphatämische Rachitis („X-linked hypophosphatemic rickets“, XLH: OMIM #307800) ist eine seltene genetische Erkrankung des Knochenstoffwechsels. Durch eine Dysregulation von „fibroblast-like-growth factor 23“ (FGF23) kommt es zu einem ausgeprägten renalen Phosphatverlust, der sich im Kindesalter mit reduziertem Längenwachstum, schwerer Rachitis und Deformitäten der Extremitäten manifestiert. Im Erwachsenenalter tragen das frühe Auftreten von Arthrose sowie chronische skeletale Schmerzen zur hohen Morbidität der Patienten bei. Seit der weltweiten Zulassung eines monoklonalen Antikörpers gegen FGF23, Burosumab, mit vielversprechenden Ergebnissen in Phase-III-Studien steht erstmals eine kausale Therapieoption für pädiatrische Patienten mit XLH zur Verfügung. Aufgrund der meist bestehenden Restsymptomatik ist die spezialisierte multidisziplinäre Versorgung der Patienten trotz Fortschritten in der Behandlung die essenzielle Grundlage in der langfristigen Betreuung der Patienten. Dabei spielt die Koordination der Behandlung innerhalb der wesentlichen medizinischen Fachgebiete eine wichtige Rolle für die optimale Therapie und Senkung der Krankheitslast. Diese Übersichtsarbeit gibt die verschiedenen Perspektiven der Versorgung von Patienten mit XLH aus Sicht von Mitgliedern eines nationalen Expertisezentrums für seltene Knochenerkrankungen wieder.

## Background

### Pathophysiology

X-linked hypophosphatemic rickets (XLH) is caused by loss-of-function of *PHEX* (phosphate-regulating gene with homology to endopeptidases on the X chromosome) and represents the most common form of hereditary hypophosphatemic rickets with a prevalence of 1/20000 newborns [1]. XLH is characterized by profound hypophosphatemia due to increased levels of a key a regulator of phosphate handling, fibroblast-like growth factor 23 (FGF23).

On a cellular level, FGF23 and its coactivator alpha-Klotho bind to fibroblast-like growth factor receptors (FGFRs) such as FGFR1 and regulate phosphate handling by two independent mechanisms [2]. In the proximal renal tubule, FGF23 inhibits phosphate reabsorption by downregulation of type II sodium/phosphate cotransporters (NaPi2a and NaPi2c)[3]. Further, an indirect reduction of intestinal resorption is exerted by downregulating 25-hydroxyvitamin D‑1*α*-hydroxylase activity and suppression of 1,25 dihydroxy-vitamin D (1,25OHD) levels [4]. Thus, endocrine FGF23 represents the major link between phosphate storage in calcified tissues and nutritional phosphate handling and allows the body to react to high phosphate intake as well as to higher phosphate needs during growth periods. While bone represents the main source of endocrine FGF23, expression can be detected in multiple tissues with a so far mostly unclear role in health and disease [5, 6].

### Clinical manifestation

In XLH, loss-of-function of the *PHEX* endopeptidase leads to an overexpression of FGF23 by unknown mechanisms. Major symptoms of XLH reflect the chronic hypophosphatemia. The development of a varus deformity of the lower limbs (bow legs) at the onset of gait is a common initial symptom of XLH. However, in toddlers it has to be distinguished from physiological bow legs, which can delay the diagnosis. Skeletal hypomineralization and rachitic growth plates typically lead to progressive varus or valgus deformities of the lower extremities. Torsional deformities, especially increased internal tibial torsion, lead to intoeing gait and impaired gait patterns. Despite optimal treatment, patients with XLH often exhibit significantly impaired longitudinal growth due to pathologic changes of the growth plates. Body height is additionally impacted by deformities of the lower limb, resulting in short stature in adulthood in a substantial number of patients with XLH. Due to relatively normal growth of the spine, disproportion is a common feature. Musculoskeletal pain and muscular weakness further impact motional function and often lead to hypomobility with an associated increase in body weight. Although less common, increased intracranial pressure and craniosynostosis because of dysregulated ossification are important symptoms and need neurological and neurosurgical care.

Dentin hypomineralization causes a high rate of endodontic infections, which can occur already in early childhood despite optimal dental care [7]. Other symptoms of the musculoskeletal apparatus such as enthesopathies, early onset of osteoarthritis and spinal deformities mainly develop in adulthood and contribute to a significant impact on quality of life and social participation. As the pathomechanism of some of these symptoms cannot be entirely explained by hypophosphatemia, treatment options often remain symptomatic.

### Patient management

**Fig. 1:**
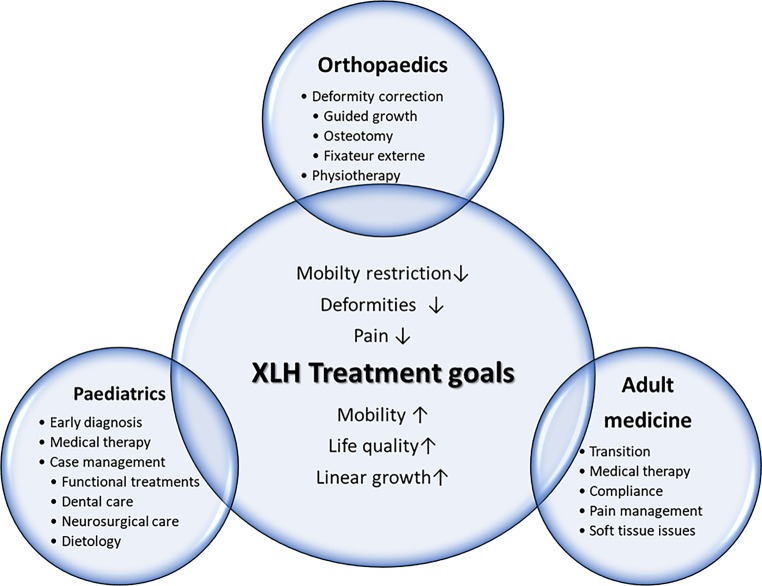
Schematic overview of treatment goals and modalities in paediatric and adult patients with X‑linked hypophosphatemic rickets (*XLH*)

The variety of symptoms in patients with XLH necessitates tight coordination of multidisciplinary patient care to optimize quality of life and reduce disease burden. This review aims to depict the current state of patient management from the perspectives of three central disciplines involved in XLH patient care, necessitating an age-related and individual treatment approach (Fig. 1).

## XLH—a paediatric perspective

Based on the recent publication of systematically developed recommendations on diagnosis and treatment of XLH, standardized management by multidisciplinary teams organized by a metabolic bone disease expert represents the mainstay of XLH patient care [8]. Due to its manifestation and onset of main symptoms during early childhood, paediatricians and paediatric orthopaedic surgeons play a special role in guiding patients and coordinating expert teams throughout the critical phase of linear growth.

### Diagnosis of XLH in children

XLH is diagnosed by clinical, radiologic and biochemical symptoms. As 20% of cases are non-familial due to de novo mutations, early diagnosis is a key challenge in paediatric patients [9]. Imaging reveals rachitic lesions with typical fraying and cupping of the growth plates. Biochemical characteristics in patients with XLH include decreased serum phosphate, increased ALP levels and elevated or inappropriately normal FGF23. The rachitic phenotype of XLH is frequently misdiagnosed as nutritional rickets, skeletal dysplasia or Blount’s disease. While subtle differences in X‑rays exist, such as lack of bone translucency with a prominent cortical compartment, XLH and nutritional rickets are often difficult to differentiate on X‑rays.

Biochemical investigations easily discriminate XLH from other diagnoses: serum phosphate levels are usually below the normal, though age-specific normal ranges have to be considered. In contrast to the most common misdiagnosis, nutritional rickets, ALP levels in XLH are lower than to be expected in severe vitamin D deficiency with severe deformities. Further, PTH levels are commonly about the upper normal range at diagnosis of XLH while being typically elevated in nutritional rickets. Inappropriately high FGF23 levels might further contribute to diagnostics but are costly and often not readily available. Regarding urine analysis, renal phosphate wasting can be confirmed by calculation of the tubular maximum reabsorption of phosphate per glomerular filtration rate (TmP/GFR) and represents a powerful and economic diagnostic measure [10]. Finally, detection of pathogenic *PHEX* mutations confirms the diagnosis of XLH, although mosaicism and intronic mutations can lead to negative results. Phenotype–genotype relation is weak, raising the importance of evaluation of family members for symptoms.

Although the onset of symptoms commonly occurs during early childhood, patients might be referred primarily to orthopaedic evaluation because of extremity deformities despite lack of nutritional rickets. Other specific symptoms such as endodental abscesses in early childhood are characteristic for XLH and should always warrant further evaluations. Thus, tight cooperation with paediatric orthopaedics, dentists and other involved disciplines are essential to minimize diagnostic delays and initiate treatment as early as possible.

### Medical treatment

Treatment should be established at best at early infancy, since early treatment is associated with better outcome [11]. Two different types of therapy exist. Conventional treatment consists of frequent dosages of oral phosphate salts combined with active vitamin D derivates such as calcitriol or alfacalcidol. Increased availability of phosphate improves bone mineralization and ameliorates skeletal symptoms [5]. The short biologic half-life of oral phosphate necessitates frequent dosages up to six times/day. Thus, compliance to treatment represents a major issue. While phosphate availability increases, FGF23 levels are further increased by treatment and serious side effects such as hyperparathyroidism and nephrocalcinosis occur frequently. The balance between optimization of skeletal symptoms and minimization side effects is challenging and requires frequent visits.

Since its registration by EMA and FDA, the monoclonal antibody burosumab has represented a novel therapeutic option. Burosumab directly binds FGF23 and directly targets the main pathomechanism of XLH and can normalize phosphate excretion. A recent phase III study could prove superiority of antibody treatment to conventional therapy regarding radiological improvement as the main study endpoint [12]. Although the patient inclusion criteria of this study limit conclusions on superiority in mildly/moderately affected individuals, burosumab broadens the therapeutic horizon especially for severely affected children and patients with insufficient response to conventional treatment. Long-term studies will be needed to judge the impact of this new therapy on major clinical burdens such as deformities and numbers of surgeries needed.

While current treatment studies focus on improvement of rachitic changes in order to prevent progression of deformities and optimize growth, a multitude of symptoms with less-defined metrics impair quality of life of patients.

Dental-related patient findings in patients with XLH are recurrent abscesses or sinus tracts associated with carious free teeth of primary and permanent dentition. Additionally, delayed tooth eruption occurs both in the primary and in the permanent dentition. Radiographically, very large pulp chambers, suggesting taurodontism, are often evident. Affected teeth show a thin enamel layer and dentinal defects as well as short roots and root resorptions in the primary dentition [13]. In contrast to systemic effects, dental symptoms appear not to be FGF23 driven but directly caused by local effects of extracellular matrix components due to impaired PHEX function [14]. Although improvement of dental abscesses has been reported under conventional therapy at least in adults [15], FGF23-blocking antibody treatment studies did not investigate dental symptoms in detail and did not reveal a reduction in dental symptoms in adverse events. The main primary treatment options in these patients are frequent dental controls and professional dental care, especially focusing on the prevention of attrition due to the fact that the structure of dental hard tissues is severely altered [13].

Bone pain is another common and challenging symptom in patients with XLH. Recent studies show prevalence of quality of life-impairing pain in 65–80% of all paediatric patients with XLH [16, 17]. Although improvement of rachitic changes by medical treatment is associated with amelioration of pain [8], further pain-relieving measures might be necessary to allow social participation and facilitate regular motor development. Involvement of specialized functional therapists may reduce symptoms and mobility impairment. In the context of overweight and obesity affecting 1/3 of children with XLH [18], any potentially achievable increase in physical activity levels is of great value in this patient group. Similar to other conditions associated with chronic pain, psychological support should be offered. Socioeconomic burdens additional to physical symptoms of the disease might hamper integration in regular schools and working life, thus social work is needed to assist patients and caretakers.

### Follow-up

Follow-up of children and adolescents with XLH includes clinical, biochemical and radiologic assessments to adjust conventional treatment and balance skeletal healing with complications such as hyperparathyroidism and nephrocalcinosis. In contrast to conventional therapy, burosumab-treated patients are targeted to reach serum phosphate values in the normal range and have to be dose-adjusted accordingly. While the reliability of the Rickets Severity Score (RSS) has recently been proven in patients with XLH [19], imaging of rachitic signs is often performed at single skeletal sites only to reduce radiation exposure. Quality of life should be assessed regularly to determine impact on everyday life and identify individual clinical burdens. Detailed recommendations on follow-up investigations are available in evidence-based consensus guidelines, ameliorating standardization of care and adaption of local follow-up protocols to the current state of clinical science [8] (follow-up scheme: Table 1).

**Table 1:** XLH follow-up plan for paediatric patients Vienna Bone and Growth Centre. (Based on [1, 7, 8])

XLH patient management plan Vienna Bone and Growth Centre	Interval
**Clinical monitoring**	
*Including height, BMI, BP, head circumference, deformity monitoring, neurological examination*	
Rapid growth phases	3‑monthly
Significant treatment changes	3‑monthly
Stable phase	6‑monthly
**Quality of life monitoring**	
PedsQL	3–6-monthly
VAS	3‑monthly
**Functional monitoring**	
6MWT	12-monthly
PEDI‑D	24-monthly
**Lab monitoring**	
*Conventional treatment*	
Ca, P, Ca/Crea ratio, ALP	3‑monthly
PTH, 25(OH)D	6‑monthly
*Burosumab*	
Initial phase: P, Ca, TmP/GFR	Week 2, 4, 8, 12
Stable phase: P, Ca, TmP/GFR	3‑monthly
PTH, 25(OH)D, 1,25(OHD), UCa/Crea	6‑monthly
**Bone imaging**	12–24-monthly
**Kidney ultrasound**	
Regular findings	24-monthly
Hypercalciuria/nephrocalcinosis	12-monthly
**Orthopaedic monitoring**	6–12 monthly
**Dental monitoring**	6‑monthly
**Cranial MRI**	If indicated
**Cardiac ultrasound**	If hypertensive

*XLH* X-linked hypophosphatemic rickets, *BMI* body mass index, *BP* blood pressure, *PedsQL* pediatric quality of life inventory, *VAS* visual analogue scale, *MWT* 6 minute walking test, *PEDI-D* pediatric evaluation of disability inventory, *Ca* calcium, *P* phosphate, *Crea* creatinine, *ALP* alkaline phosphatase, *PTH* parathyroid hormone, *TmP/GFR* ratio of the maximum rate of tubular phosphate reabsorption to glomerular filtration rate, *25(OH)D* 25-hydroxycholecalciferol, *1,25(OHD)* 1,25-dihydroxycholecalciferol, *UCa/Crea* urinary calcium to creatinine ratio

## XLH—an adult osteological perspective

XLH in adults is associated with several musculoskeletal symptoms including osteomalacia, enthesopathy and muscle weakness. Almost all adults report bone or joint pain, and nearly every second adult XLH patient has already sustained a fracture [17]. Extra-skeletal manifestations include dental complications (e.g. periodontitis) and hearing loss. Consequently, quality of life in XLH patients is often severely impaired. Typical radiographic features in adult patients with XLH are pseudofractures and osteoarthritis of the spine and joints. Calcifications of ligaments and enthesopathies can also be detected by X‑ray examinations.

### Diagnosis of XLH in adults

Based on recent recommendations for the management of X‑linked hypophosphatemia, the diagnosis of XLH in adults should be considered in case of typical clinical or radiological signs and low levels of serum phosphate as well as renal phosphate wasting [8]. A positive family history for XLH is indicative. The clinical diagnosis can be confirmed by genetic testing. Usually alkaline phosphatase (ALP) and intact FGF23 levels are increased. However, FGF23 levels can be normal in XLH, and high FGF23 levels can also be found in other diseases [8]. Differential diagnosis of high serum FGF23 levels include fibrous dysplasia, autosomal dominant or recessive hypophosphatemic rickets and Raine syndrome [20].

### Management of adult XLH patients

Management of adult XLH patients includes regular laboratory and radiological examinations as well as assessment of pain, muscle strength, mobility and treatment response. An important part of adult XLH care is the transition from the paediatric clinic to the department of internal medicine. Specific requirements and individual needs of adult XLH patients must be observed. Specialists for internal medicine, rehabilitation medicine, dentistry and orthopaedic surgery are therefore involved in the management of adult XLH patients. An XLH support group was recently founded in Austria to give patients the opportunity to exchange their experiences and to facilitate their lives (www.phosphatdiabetes.at).

#### Medical treatment

Medical treatment in adult XLH patients is quite similar to that in children with XLH, including phosphate and active vitamin D substitution. Conventional treatment in adults is only recommended in symptomatic patients [1]. In asymptomatic adult XLH patients, treatment does not seem to be beneficial. However, based on recent recommendations, treatment should also be considered in case of planned orthopaedic or dental surgery or evidence of osteomalacia [8].

Although a positive effect of conventional therapy on pain, osteomalacia and oral health has been suggested, phosphate and active vitamin D substitution do not prevent the occurrence of enthesopathies [21]. Anti-resorptive drugs including bisphosphonates and denosumab must be avoided. For teriparatide, an osteoanabolic agent, there are no data available in XLH. Calcium supplementation is not recommended.

In Europe, burosumab—a anti FGF23 antibody—is currently approved for treatment of children with XLH. However, promising data have already been published for adults. A normalization of phosphate levels and an improvement in fracture healing was shown under anti-FGF23 antibody therapy [22]. Treatment continuation of burosumab improved pain, stiffness and physical function in adult patients with XLH [23].

The effect of burosumab on osteomalacia was published recently. In this paired bone biopsy study, 48 weeks of burosumab treatment resulted in an improvement of osteomalacia-related histomorphometric parameters [24].

#### Follow-up of adult XLH patients

Follow-up examinations should include ALP, calcium, phosphate, creatinine, parathyroid hormone (PTH) and 25(OH) vitamin D. Moreover, screening for skeletal manifestations as well as blood pressure, dental disease, pain, fatigue and muscular strength are recommended [8]. In patients receiving therapy, a renal ultrasound should be performed in order to exclude kidney stone disease [1]. Dental-related symptoms, as mentioned in the paediatric perspective, urge for frequent checks at specialized centres to minimize tooth loss and dental abscesses. In contrast to other bone diseases, bone mineral density (BMD) measurements by DXA scanning do not play a major role in the diagnosis or follow-up of adult XLH patients. A high trabecular BMD can be observed in most XLH patients. In contrast, cortical BMD is usually low [25]. Serum FGF23 levels are useful in untreated individuals but cannot guide treatment decisions in patients receiving adequate therapy.

### Differential diagnosis of low phosphate levels in adults

It must be considered that low phosphate levels and skeletal symptoms in adults might have several reasons. Tumour-induced osteomalacia (TIO) is caused mainly by mesenchymal tumours and is associated with low phosphate levels due to paraneoplastic FGF23 overproduction [26]. In general, TIO is curable if the tumour can be removed [27]. In case of unresectable or unidentifiable tumour, a conservative treatment should be initiated. A case of TIO due to meningioma and effective burosumab treatment was published recently [28]. Moreover, several drugs such as adefovir or intravenous iron therapy can lead to hypophosphatemia and similar symptoms to XLH [29, 30]. Therefore, TIO, drug-induced hypophosphatemia and other forms of osteomalacia and renal tubular phosphate wasting must also be considered in adult patients with low phosphate levels.

## XLH—a paediatric orthopaedic perspective

The paediatric orthopaedic treatment of patients with XLH can be a challenging task with the goal of prevention and treatment of deformity. Decision-making for the right surgical treatment and its optimal timing is more complex in patients with XLH than in other paediatric orthopaedic aetiologies.

### Diagnostics

An accurate physical examination with focus on gait, rotational profile of the lower legs and range of motion of the joints is the cornerstone of further diagnostic measures. Basic radiographic examination is obtained with a long-leg standing X‑ray. This view enables us to detect joint abnormalities, leg length discrepancies, multiapical bone deformities and deviation of the axis (varus or valgus). Furthermore, the lateral view of the lower leg is important to detect eventual masked deformities. As torsional deformities of bones are quite common in patients with XLH torsion, MRI (preferred method) or CT can be helpful for preoperative planning. Additional information on compensation mechanisms can be gained using 3D gait analysis.

**Fig. 2:**
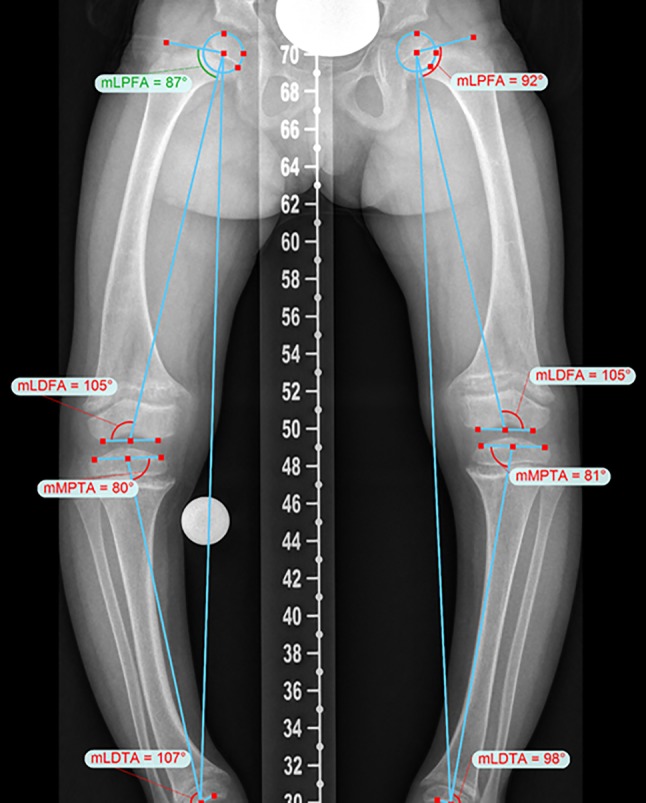
Accurate preoperative deformity analysis allows an optimizes surgical treatment approach in patients with X‑linked hypophosphatemic rickets

Surgical treatment plans need individual, careful and comprehensive preoperative deformity analysis. Multiple angles on standing X‑rays have to be measured to choose the right surgical technique and the accurate amount of correction (Fig. 2; [31, 32]). Different software programs such as Trauma CAD software (Voyant Healt, Petach-Tikva, Israel) or the Bone Ninja (Rubin Institute for Advanced Orthopedics, Sinai Hospital of Baltimore; Baltimore, MD, USA) app can help to analyse the deformity and make our treatment plans more understandable for our patients.

### Orthopaedic management

Adequate basic medical treatment and physiotherapy are the best conservative treatment options. Pain can be a sign for inadequate medication and does not necessarily correlate with bony deformity, especially in younger children. Critical observation with regular clinical and radiographic follow-up in cases of borderline deformity are the main paediatric orthopaedic activities during growth.

Historically, long leg braces were thought to prevent deformity in children with XLH. However, there is no scientific evidence to support the use of braces to guide growth in children with XLH [33]. The paediatric orthopaedic surgeon needs a comprehensive knowledge of physiological and pathological growth, of deformity analysis and deformity correction, and needs to be trained in the different surgical procedures. Various surgical procedures with differences in the amount of achievable correction, complication profile and quality of life are used to treat lower limb deformity in children with XLH.

### Surgical treatment options

Guided growth with hemiepiphysiodesis is a minimally invasive treatment option for mild deformities of the lower limbs. Several studies showed favourable results in children with healthy and pathological physis [34–36]. Depending on the deformity, the growth plate is temporarily blocked using a small plate with two screws on the medial or lateral distal femur or proximal tibia. However, torsional deformities cannot be treated with this method. Children with XLH have to be younger for this technique, due to reduced growth velocity and therefore reduced correction potential. The procedure requires open growth plates and a second operation to remove the implant after full correction.

Promising postoperative results in children with XLH treated with hemiepiphysiodesis have been reported [33, 37, 38] and combined with careful metabolic control, this method can prevent the need for osteotomies [38]. However, a higher failure rate in children with XLH has been described [39]; nevertheless, the method can be repeated safely.

A more invasive method to correct lower limb deformities are osteotomies with different types of fixation. Deformities can be corrected acutely or gradually. In acute deformity correction the osteotomy has to be stabilized using plates and screws or intramedullary nails. In younger children, wires in combination with a cast can be used. More complex deformities need to be corrected gradually. A unilateral or ring fixator can be used for external fixation and gradual lengthening and/or axis correction. Even most severe deformities can be treated safely with an external ring fixator. It takes several months until the bone is perfectly healed and the frame is removed. A more convenient method uses intramedullary lengthening nails which allow deformity correction and lengthening of bones. Technical limitations of these intramedullary implants are the severe bowing of the femora and tibiae often seen in XLH patients.

Recurrence of deformity after surgery can occur irrespective of the method used in children with XLH, and needs to be treated adequately. Rehabilitation after surgery in patients with XLH is a multidisciplinary effort, especially when external fixators are used. Adequate metabolic control, appropriate pain management, devices for mobility and individual physiotherapy by specially trained physiotherapists are necessary to achieve high patient satisfaction and best postoperative results. Psychologic counselling and the possibility to attend school during the time of hospitalization are extraordinarily important in a paediatric surgical treatment setting.

## Conclusion

In a multisystemic disorder such as XLH, management and integration of specialized subdisciplines is of great importance to minimize long-term sequalae and optimize quality of life. Recent advances in pharmacologic therapy of XLH as well as refined orthopaedic techniques contribute to improve skeletal symptoms. Nevertheless, most patients remain symptomatic and more specific treatment approaches, especially for life-impairing symptoms such as bone pain, enthesopathies and dental problems, are needed. The formation of multidisciplinary expert centres, as well as the implementation of individualized patient care and transnational clinical studies will further help to target remaining challenges in the management of XLH.

## References

[1] Carpenter TO, Imel EA, Holm IA, Jan de Beur SM, Insogna KL (2011). A clinician’s guide to X-linked hypophosphatemia. J Bone Miner Res.

[2] Gattineni J, Twombley K, Goetz R, Mohammadi M, Baum M (2011). Regulation of serum 1,25(OH)2Vitamin D3 levels by fibroblast growth factor 23 is mediated by FGF receptors 3 and 4. Am J Physiol.

[3] Segawa H, Kawakami E, Kaneko I, Kuwahata M, Ito M, Kusano K (2003). Effect of hydrolysis-resistant FGF23-R179Q on dietary phosphate regulation of the renal type-II Na/Pi transporter. Pflugers Arch.

[4] Shimada T, Hasegawa H, Yamazaki Y, Muto T, Hino R, Takeuchi Y (2004). FGF-23 is a potent regulator of vitamin D metabolism and phosphate homeostasis. J Bone Miner Res.

[5] Raimann A, Ertl DA, Helmreich M, Sagmeister S, Egerbacher M, Haeusler G (2013). Fibroblast growth factor 23 and Klotho are present in the growth plate. Connect Tissue Res.

[6] Faul C (2012). Fibroblast growth factor 23 and the heart. Curr Opin Nephrol Hypertens.

[7] Linglart A, Biosse-Duplan M, Briot K, Chaussain C, Esterle L, Guillaume-Czitrom S (2014). Therapeutic management of hypophosphatemic rickets from infancy to adulthood. Endocr Connect.

[8] Haffner D, Emma F, Eastwood DM, Duplan MB, Bacchetta J, Schnabel D (2019). Clinical practice recommendations for the diagnosis and management of X-linked hypophosphataemia. Nat Rev Nephrol.

[9] Whyte MP, Schranck FW, Armamento-Villareal R (1996). X-linked hypophosphatemia: a search for gender, race, anticipation, or parent of origin effects on disease expression in children. J Clin Endocrinol Metab.

[10] Brodehl J, Krause A, Hoyer PF (1988). Assessment of maximal tubular phosphate reabsorption: comparison of direct measurement with the nomogram of Bijvoet. Pediatr Nephrol.

[11] Mäkitie O, Doria A, Kooh SW, Cole WG, Daneman A, Sochett E (2003). Early treatment improves growth and biochemical and radiographic outcome in X-linked hypophosphatemic rickets. J Clin Endocrinol Metab.

[12] Imel EA, Glorieux FH, Whyte MP, Munns CF, Ward LM, Nilsson O (2019). Burosumab versus conventional therapy in children with X-linked hypophosphataemia: a randomised, active-controlled, open-label, phase 3 trial. Lancet.

[13] Sabandal MMI, Robotta P, Bürklein S, Schäfer E (2015). Review of the dental implications of X-linked hypophosphataemic rickets (XLHR). Clin Oral Invest.

[14] Coyac BR, Hoac B, Chafey P, Falgayrac G, Slimani L, Rowe PS (2018). Defective mineralization in X-linked hypophosphatemia dental pulp cell cultures. J Dent Res.

[15] Econs MJ (2015). Conventional therapy in adults with XLH improves dental manifestations, but not enthesopathy. J Clin Endocrinol Metab.

[16] Emma F, Cappa M, Antoniazzi F, Bianchi ML, Chiodini I, Eller Vainicher C (2019). X-linked hypophosphatemic rickets: an Italian experts’ opinion survey. Ital J Pediatr.

[17] Skrinar A, Dvorak-Ewell M, Evins A, Macica C, Linglart A, Imel EA (2019). The lifelong impact of X-linked hypophosphatemia: results from a burden of disease survey. J Endocr Soc.

[18] Zhukouskaya V, Lambert A-S, Rothenbuhler A, Colao A, Di Somma C, Kamenicky P (2019). SAT-259 natural history of anthropometric parametres of obesity in children affected by X-linked hypophosphatemia: longitudinal obserbational study. J Endocr Soc.

[19] Thacher TD, Pettifor JM, Tebben PJ, Creo AL, Skrinar A, Mao M (2019). Rickets severity predicts clinical outcomes in children with X-linked hypophosphatemia: utility of the radiographic rickets severity score. Bone.

[20] Huang X, Jiang Y, Xia W (2013). FGF23 and phosphate wasting disorders. Bone Res.

[21] Connor J, Olear EA, Insogna KL, Katz L, Baker S, Kaur R (2015). Conventional therapy in adults with X-linked hypophosphatemia: effects on enthesopathy and dental disease. J Clin Endocrinol Metab.

[22] Insogna KL, Briot K, Imel EA, Kamenický P, Ruppe MD, Portale AA (2018). A randomized, double-blind, placebo-controlled, phase 3 trial evaluating the efficacy of Burosumab, an anti-FGF23 antibody, in adults with X-linked Hypophosphatemia: week 24 primary analysis. J Bone Miner Res.

[23] Portale AA, Carpenter TO, Brandi ML, Briot K, Cheong HI, Cohen-Solal M (2019). Continued beneficial effects of Burosumab in adults with X-linked hypophosphatemia: results from a 24-week treatment continuation period after a 24-week double-blind placebo-controlled period. Calcif Tissue Int.

[24] Insogna KL, Rauch F, Kamenický P, Ito N, Kubota T, Nakamura A (2019). Burosumab improved histomorphometric measures of osteomalacia in adults with X-linked hypophosphatemia: a phase 3, single-arm, international trial. J Bone Miner Res.

[25] Cheung M, Roschger P, Klaushofer K, Veilleux L-N, Roughley P, Glorieux FH (2013). Cortical and trabecular bone density in X-linked hypophosphatemic rickets. J Clin Endocrinol Metab.

[26] Yin Z, Du J, Yu F, Xia W (2018). Tumor-induced osteomalacia. Osteoporos Sarcopenia.

[27] Mishra SK, Kuchay MS, Sen IB, Garg A, Baijal SS, Mithal A (2019). Successful management of tumor-induced osteomalacia with radiofrequency ablation: a case series. JBMR Plus.

[28] Day AL, Gutiérrez OM, Guthrie BL, Saag KG (2019). Burosumab in tumor-induced osteomalacia: a case report. Joint Bone Spine.

[29] Koda R, Tsuchida M, Iino N, Narita I (2019). Hypophosphatemic osteomalacia associated with adefovir-induced Fanconi syndrome initially diagnosed as diabetic kidney disease and vitamin D deficiency. Intern Med.

[30] Bartko J, Roschger P, Zandieh S, Brehm A, Zwerina J, Klaushofer K (2018). Hypophosphatemia, severe bone pain, gait disturbance, and fatigue fractures after iron substitution in inflammatory bowel disease: a case report. J Bone Miner Res.

[31] Paley D, Herzenberg JE, Tetsworth K, McKie J, Bhave A (1994). Deformity planning for frontal and sagittal plane corrective osteotomies. Orthop Clin North Am.

[32] Paley D, Tetsworth K (1992). Mechanical axis deviation of the lower limbs. Preoperative planning of multiapical frontal plane angular and bowing deformities of the femur and tibia. Clin Orthop Relat Res.

[33] Novais E, Stevens PM (2006). Hypophosphatemic rickets: the role of hemiepiphysiodesis. J Pediatr Orthop.

[34] Stevens PM, Klatt JB (2008). Guided growth for pathological physes: radiographic improvement during realignment. J Pediatr Orthop.

[35] Saran N, Rathjen KE (2010). Guided growth for the correction of pediatric lower limb angular deformity. J Am Acad Orthop Surg.

[36] Danino B, Rödl R, Herzenberg JE, Shabtai L, Grill F, Narayanan U (2018). Guided growth: preliminary results of a multinational study of 967 physes in 537 patients. J Child Orthop.

[37] Sharkey MS, Grunseich K, Carpenter TO (2015). Contemporary medical and surgical management of X-linked hypophosphatemic rickets. J Am Acad Orthop Surg.

[38] Horn A, Wright J, Bockenhauer D, Van’t Hoff W, Eastwood DM (2017). The orthopaedic management of lower limb deformity in hypophosphataemic rickets. J Child Orthop.

[39] Masquijo JJ, Firth GB, Sepúlveda D (2017). Failure of tension band plating. J Pediatr Orthop B.

